# The Role of Olorofim in the Treatment of Filamentous Fungal Infections: A Review of In Vitro and In Vivo Studies

**DOI:** 10.3390/jof10050345

**Published:** 2024-05-10

**Authors:** Aliosha Feuss, Marie-Elisabeth Bougnoux, Eric Dannaoui

**Affiliations:** 1 Mycology Unit, Necker-Enfants Malades University Hospital, Assistance Publique des Hôpitaux de Paris (AP-HP), 75015 Paris, France; dr.feuss@gmail.com (A.F.); marie-elisabeth.bougnoux@aphp.fr (M.-E.B.); 2 Faculty of Medicine, Paris Cité University, Necker Campus, 75015 Paris, France; 3 DYNAMYC UR 7380, Faculty of Medicine, Paris-Est Créteil University (UPEC), 94000 Créteil, France

**Keywords:** olorofim, orotomides, antifungal, azole resistance, filamentous fungi, invasive fungal disease

## Abstract

Invasive fungal infections have recently been recognized by the WHO as a major health, epidemiological, and economic issue. Their high mortality rates and the emergence of drug resistance have driven the development of new molecules, including olorofim, an antifungal belonging to a new family of compounds, the orotomides. A review was conducted on the PubMed database and the ClinicalTrials.gov website to summarize the microbiological profile of olorofim and its role in the treatment of filamentous fungal infections. Twenty-four articles were included from the search and divided into two groups: an “in vitro” group focusing on minimum inhibitory concentration (MIC) results for various fungi and an “in vivo” group evaluating the pharmacokinetics (PK), pharmacodynamics (PD), efficacy, and tolerability of olorofim in animal models of fungal infection and in humans. Olorofim demonstrated in vitro and in vivo activity against numerous filamentous fungi, including azole-resistant *Aspergillus fumigatus*, various dermatophytes, and endemic and dimorphic fungi. in vitro results showed higher MICs for certain *Fusarium* species and dematiaceous fungi *Alternaria alternata* and *Exophiala dermatitidis*; further in vivo studies are needed. Published PK-PD data in humans are limited. The results of the ongoing phase III clinical trial are eagerly awaited to evaluate olorofim’s clinical impact.

## 1. Introduction

The rise of fungal infections presents a distinctive challenge in the medical realm. Unlike bacteria or viruses, these eukaryotic organisms share metabolic pathways with humans, necessitating a delicate approach in antifungal treatment development to minimize adverse effects on the latter [1]. Severe invasive fungal infections (IFIs), significantly impacting vulnerable populations like immunocompromised patients, neonates, and intensive care unit patients, have become a growing concern. Recognized by the World Health Organization as a critical threat, the filamentous fungus *Aspergillus fumigatus*, the principal agent responsible for aspergillosis, is increasingly showing resistance to conventional treatments like voriconazole [2]. In this context, the orotomides, exemplified by olorofim, stand as a beacon of hope. Initially developed by F2G Limited, these antifungals represent the first novel class in the field in 20 years. The discovery of olorofim came after a meticulous evaluation involving structure–activity relationships, and subsequent evaluations determined its pharmacokinetics and efficacy in animal models. While its mechanism of action was initially unknown, a complex microbiological and genetic approach identified dihydroorotate dehydrogenase (DHODH) as the target [3]. By specifically inhibiting the fungal DHODH, olorofim disrupts essential processes in pyrimidine synthesis, DNA/RNA formation, protein synthesis, and cell cycle regulation. Notably, olorofim displayed a dual action on *A. fumigatus*, proving fungistatic initially while rapidly transitioning to a time-dependent fungicidal effect upon prolonged exposure [4,5]. These observations underscore the potential of olorofim in reshaping the landscape of antifungal treatment, although further research is necessary to determine its applicability to a broader spectrum of fungal species beyond *A. fumigatus*. This article aims to determine the role of olorofim in the treatment of IFIs through a review of in vitro and in vivo studies published to date.

## 2. Materials and Methods

Article selection was conducted from 1 October 2022 to 31 March 2023 within the PubMed^®^ and www.clinicaltrials.gov databases. Keywords “F901318” and “olorofim” were used as searched in titles and abstracts on PubMed^®^ and in titles and interventions on www.clinicaltrials.gov. All studies meeting the keyword search criteria during the screening step were explored. Initially, articles on yeasts, articles on the general antifungal pipeline, and clinical studies with no published results were excluded. Subsequently, during the eligibility step, articles on in vitro studies without minimal inhibitory concentration (MIC) results, articles with results on one isolated strain of a defined species, articles with results from strains used in other studies, and in vivo studies lacking pharmacokinetic (PK), pharmacodynamic (PD), efficacy, or tolerability results were excluded.

## 3. Results

**Figure 1:**
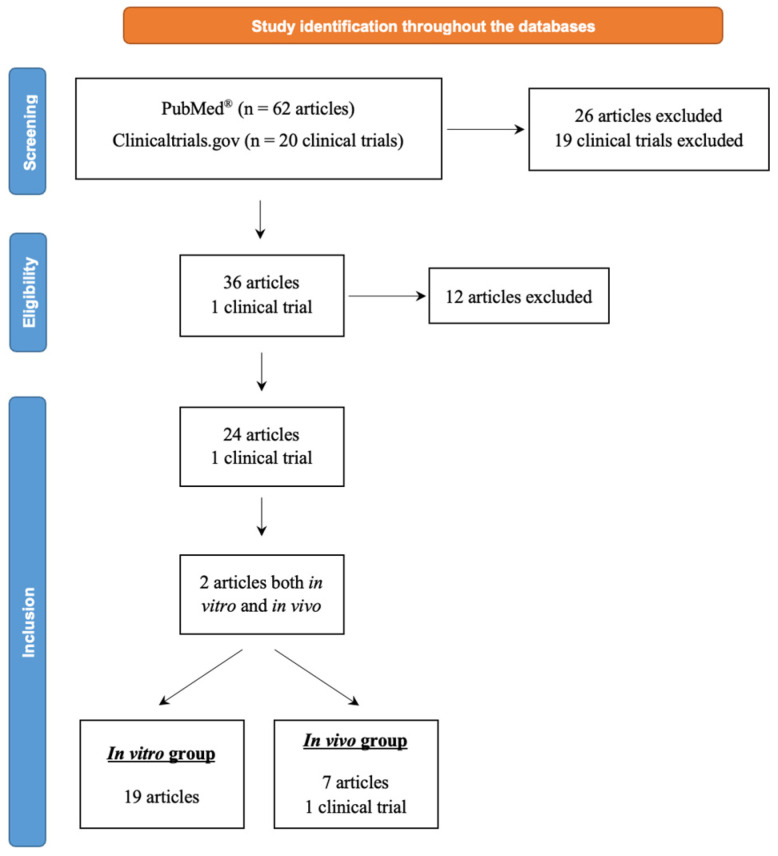
Flux diagram of the olorofim studies selection process for the review.

As shown in Figure 1, a total of 62 articles were identified during the screening step, 36 of which were eligible for inclusion in this review. These articles were published between 2016 and 2023 by research teams from every continent. Ultimately, 24 articles were included and divided into two groups: an “in vitro” group focusing on olorofim MIC results for various filamentous and dimorphic fungi and an “in vivo” group with available PK, PD, efficacy, or tolerability data using animal models of invasive fungal infections. The one clinical trial whose results are available on www.clinicaltrials.gov was included in the second group. Furthermore, it was found that two articles belonged in both the in vitro and in vivo groups.

### 3.1. In Vitro Studies

Table 1 summarizes the MICs observed for various genera and species of filamentous and dimorphic fungi as determined by the EUCAST or CLSI standard methods.

As previously described, olorofim’s development was driven by its potent antifungal activity against azole-resistant strains of *A. fumigatus*. Five studies explored the MIC distribution of a total of 1496 wild-type *A. fumigatus* strains and 276 resistant strains with all mechanisms combined [6,7,8,9,10]. The observed geometric mean MICs (GMICs) ranged from 0.025 to 0.053 mg/L and 0.031 to 0.058 mg/L for wild-type and resistant strains, respectively. MIC_90_ values ranged from 0.031 to 0.125 mg/L for the wild-type strains and from 0.063 to 0.125 mg/L for the azole-resistant strains. Five research teams investigated olorofim’s MICs for species complexes other than *Fumigati*. Those included *Flavi*, *Nidulantes*, *Nigri*, *Terrei*, and *Usti* [6,7,8,11,12]. In Astvad et al.’s study, only strains from the *Usti* section displayed slightly higher MICs compared to those observed in *A. fumigatus*, ranging from 0.06 to 0.5 mg/L for some strains in a small population of seven.

Olorofim has been tested on other genera than *Aspergillus*. In four studies involving *Scedosporium* spp., the observed GMIC for 170 strains ranged from 0.009 to 0.193 mg/L, with MIC_90_ values ranging from 0.03 to 0.5 mg/L [13,14,15,16]. Limited data exist on the genus *Fusarium*, and the results seem species-dependent. For instance, in the studies by Jørgensen et al. and Astvad et al., the MICs for four strains of *Fusarium proliferatum* were low, ranging from 0.03 to 0.06 mg/L. However, for *Fusarium solani* and *Fusarium oxysporum*, MICs were >1 mg/L [7,8]. The study with the largest number of strains belonging to these two species complexes was conducted by Badali et al. [17]. Species belonging to the *F. oxysporum* complex seemed to have lower MICs compared to those in the *F. solani* complex, with GMIC values of 0.515 mg/L and at least 4 mg/L, respectively, in agreement with the results of Georgacopoulos et al. [9]. Olorofim was also tested against dematiaceous fungi *Alternaria alternata* [16] and *Exophiala dermatitidis* [16,18]. The results were modest, especially for the *E. dermatitidis*, with a MIC_90_ > 4 mg/L, comparable to that of certain azoles and amphotericin B. In vitro activity was also studied by Lim et al. on 21 strains of *Madurella mycetomatis*, coming mainly from Sudan [19]. The observed MIC_90_ was 0.063 mg/L, lower than that of itraconazole (0.125 mg/L). Wiederhold et al. tested some strains of *Microascus*/*Scopulariopsis* spp., *Rasamsonia argillacea* species complex, several strains of *Penicillium* spp., and a few strains of *Talaromyces* spp. (including 7 *Talaromyces marneffei*) [20]. The observed MICs were low, even very low for *R. argillacea* and *T. marneffei*, with a GMIC around 0.008 mg/L. Zhang et al. corroborated these findings [21]. Olorofim was also tested against other dimorphic fungi such as *Sporothrix brasiliensis* by Bombassaro et al. and *Coccidioides* spp. by Wiederhold et al. [22,23]. For *S. brasiliensis*, the GMIC was 0.026 mg/L among 52 strains; as for *Coccidioides* spp., the GMIC was 0.011 mg/L among 59 strains in total.

Lastly, olorofim was tested in vitro against certain dermatophytes, including *Trichophyton indotineae*, a recalcitrant species to conventional antifungal treatments and the most represented species in Singh et al.’s study [16]. It showed a GMIC of 0.01 mg/L among a total of 46 strains. Seven strains of *Trichophyton tonsurans* and three strains of *Trichophyton rubrum* also exhibited low MICs, ranging from 0.03 to 0.125 mg/L, and of 0.06 mg/L, respectively.

**Table 1:** In vitro olorofim susceptibility against various filamentous and dimorphic fungi.

Genus and Species	Reference	Method	Number of Strains	GM MIC (mg/L)	MIC_50_ (mg/L)	MIC_90_ (mg/L)
***Aspergillus* spp.**						
*Aspergillus alabamensis* (*Terrei* complex)	[11]	CLSI	8		0.016	0.03
*Aspergillus alliaceus* (*Flavi* complex)	[12]	EUCAST	20	0.024	0.03	0.03
*Aspergillus aureoterreus* (*Terrei* complex)	[12]	EUCAST	3	0.015	ND	ND
*Aspergillus calidoustus* *(Usti complex)*	[6]	EUCAST	25		0.25	0.5
	[12]	EUCAST	20	0.098	0.125	0.25
*Aspergillus carneus* (*Terrei* complex)	[12]	EUCAST	3	0.019	ND	ND
*Aspergillus citrinoterreus* *(Terrei complex)*	[12]	EUCAST	5	0.015	ND	ND
	[11]	CLSI	27		0.016	0.03
*Aspergillus flavus* SC	[6]	EUCAST	10		0.03	0.06
	[7]	EUCAST	12	0.050		
	[8]	EUCAST	48	0.029	0.03	0.06
*Aspergillus fumigatiaffinis* (*Fumigati* complex)	[12]	EUCAST	10	0.016	0.016	0.016
*Aspergillus fumigatus stricto sensu* azole-resistant	[6]	EUCAST	133			
			25 TR34/L98H		0.125	0.125
			25 TR46/Y121F/T289A		0.125	0.125
			33 point mutations		0.03	0.06
			50 other mechanism		0.06	0.125
	[7]	EUCAST	22	0.042		
	[8]	EUCAST	112	0.058	0.06	0.125
	[9]	CLSI	5 TR34/L98H		0.008	
	[10]	EUCAST	4	0.031		
*Aspergillus fumigatus* wild-type	[6]	EUCAST	10		0.06	0.125
	[7]	EUCAST	213	0.037		
	[8]	EUCAST	920	0.053	0.06	0.06
	[9]	CLSI	246		0.008	
	[10]	EUCAST	107	0.025	0.03	0.03
*Aspergillus hiratsukae* (*Fumigati* complex)	[12]	EUCAST	7	0.016	0.016	0.016
*Aspergillus hortai* (*Terrei* complex)	[12]	EUCAST	2	0.015	ND	ND
	[11]	CLSI	13		0.016	0.03
*Aspergillus insuetus* (*Usti* complex)	[12]	EUCAST	3	0.196	ND	ND
*Aspergillus keveii* (*Usti* complex)	[12]	EUCAST	2	0.085	ND	ND
*Aspergillus lentulus* (*Fumigati* complex)	[12]	EUCAST	20	0.017	0.016	0.03
*Aspergillus nidulans* SC	[6]	EUCAST	10		0.125	0.125
	[8]	EUCAST	17	0.069	0.06	0.125
*Aspergillus niger* SC	[7]	EUCAST	17	0.052		
	[8]	EUCAST	129	0.08	0.06	0.125
*Aspergillus ochraceus* (*Circumdati* complex)	[12]	EUCAST	10	0.02	0.016	0.03
*Aspergillus sclerotiorum* (*Circumdati* complex)	[12]	EUCAST	5	0.017	ND	ND
*Aspergillus terreus* SC	[7]	EUCAST	5	0.022		
	[8]	EUCAST	64	0.023	0.03	0.06
*Aspergillus terreus stricto sensu*	[11]	CLSI	42		0.004	0.008
*Aspergillus thermomutatus* (*Fumigati* complex)	[8]	EUCAST	11	0.057	0.06	0.125
	[12]	EUCAST	10	0.016	0.016	0.016
*Aspergillus tubingensis* (*Nigri* complex)	[6]	EUCAST	25		0.03	0.06
	[8]	EUCAST	18	0.087	0.06	0.125
	[12]	EUCAST	20	0.051	0.06	0.06
*Aspergillus udagawae* (*Fumigati* complex)	[12]	EUCAST	10	0.016	0.016	0.016
**Other fungi**						
*Alternaria alternata*	[16]	CSLI	32	2	2	2
*Coccidioides immitis*	[23]	CLSI	21	0.009	<0.008	0.016
*Coccidioides posadasii*	[23]	CLSI	24	0.009	<0.008	0.016
*Exophiala dermatitidis*	[18]	EUCAST	10	ND		>4
*Fusarium oxysporum* SC	[17]	CLSI	45	0.515	0.5	4
	[9]	CLSI	5		2	
*Fusarium solani* SC	[17]	CLSI	16	ND	>4	>4
	[9]	CLSI	11		>2	
*Lomentospora prolificans*	[13]	CLSI	7	0.168	0.125	0.25
	[14]	CLSI	30	0.26	0.25	0.25
	[15]	EUCAST	30	0.115	0.125	0.25
	[18]	EUCAST	20	0.202		
*Madurella mycetomatis*	[19]	Modified CLSI	21		0.016	0.06
*Microascus/Scopulariopsis* spp.	[20]	CLSI	59	0.033	0.03	0.125
*penicillium* spp.	[20]	CLSI	28	0.021	0.016	0.25
*pseudallescheria ellipsoidea* (*S. apiospermum* complex)	[15]	EUCAST	10	0.052	0.06	0.125
*Rasamsonia argillacea* SC	[18]	EUCAST	23	ND		<0.008
	[20]	CLSI	46	ND	<0.008	<0.008
*Scedosporium apiospermum* *stricto sensu*	[13]	CLSI	43	0.079	0.06	0.25
	[14]	CLSI	10	0.016	0.125	0.25
	[15]	EUCAST	30	0.05	0.06	0.125
	[16]	CLSI	11	0.009	0.004	0.03
*Scedosporium aurantiacum*	[13]	CLSI	6	0.193	0.125	0.5
	[15]	EUCAST	20	0.13	0.125	0.25
*Scedosporium boydii* (*S. apiospermum* complex)	[13]	CLSI	15	0.046	0.06	0.06
	[15]	EUCAST	30	0.04	0.03	0.125
*Scedosporium dehoogii*	[13]	CLSI	2	ND	0.125	0.5
	[15]	EUCAST	3	0.095	ND	ND
*Sporothrix brasiliensis*	[22]	CLSI	52	0.026	0.03	0.125
*Talaromyces* spp.	[20]	CLSI	27 (of which 7 *T. marneffei*)	0.008	<0.008	0.016
*Talaromyces marneffei*	[21]	CLSI	32	0.0005	0.0005	0.0005
*Trichophyton indotine* *ae*	[16]	CLSI	46	0.01	0.008	0.03
*Trichophyton rubrum*	[8]	EUCAST	24	0.048	0.06	0.06

CLSI: Clinical and Laboratory Standards Institute. EUCAST: European Committee on Antimicrobial Susceptibility Testing. ND: not determined. SC: species complex.

### 3.2. In Vivo Studies

Table 2 summarizes the pharmacokinetic and pharmacodynamic data from the literature, while Table 3 compiles the survival studies conducted in animal models of invasive fungal infection. Their full versions can be found in the Supplementary Materials.

Olorofim demonstrates time-dependent antifungal activity and high protein binding, as first shown by Hope et al. [24]. The main pharmacodynamic parameter was serum galactomannan (GM) levels at 78 h post-infection in a neutropenic murine model of invasive pulmonary aspergillosis. Interestingly, when a daily dose of 24 mg/kg was divided into three administrations, complete suppression of serum galactomannan was observed in infected mice compared to the placebo *(p* = 0.024). No difference was found between the once-daily administration and the placebo. These results held true for mice infected with both wild-type *A. fumigatus* strains and T_34_/L98H strains. To further explore this time-dependent effect, the team examined the relationship between olorofim’s maximum plasma concentration (C_max_), the area under the concentration–time curve (AUC), the minimum plasma concentration (C_min_), and the MIC of the tested strains. Serum galactomannan at 78 h and the area under the galactomannan–time curve (AUC-GM) were used as the pharmacodynamic parameters. No correlation was found for the C_max_/MIC ratio nor the AUC/MIC ratio. However, a strong correlation was observed for the C_min_/MIC ratio (*r*^2^ = 0.983 for GM and *r*^2^ = 0.998 for the AUC-GM) and for the time spent above a concentration of 1 mg/L “*T* > 1 mg/L” (*r*^2^ = 0.996 for GM and *r*^2^ = 1.00 for the AUC-GM). These results confirm the time-dependent nature of the antifungal effect. Additionally, a 27% reduction in AUC-GM was observed for a C_min_/MIC ratio of 9.1, corresponding to a C_min_ of 0.27 mg/L. These findings align with those of Negri et al. in an invasive sinopulmonary *A. flavus* infection model [25]. A decrease in AUC-GM was observed for an average C_min_/MIC ratio of 13.38 (range 9–19). Lackner et al. demonstrated antifungal activity for a C_min_ of 0.3 mg/L in a neutropenic murine model of systemic *A. terreus* infection [11]. In a neutropenic murine model of systemic *A. fumigatus* infection, Seyedmousavi et al. found C_min_ values between 0.1 and 0.3 mg/L, consistent with the previous studies [26].

The same research teams conducted survival studies to evaluate the in vivo efficacy of olorofim in treating invasive fungal infections in neutropenic animal models. Hope et al. and Negri et al. assessed survival on day 10 and pulmonary histology on day 3 of infection in neutropenic murine models infected with *A. fumigatus* and *A. flavus*, respectively [24,25]. Both teams found no difference in survival between treatment with 24 mg/kg/day administered once and the placebo. In Hope et al.’s study, the median survival time was approximately 8 days for the 8 mg/kg/q8h IV group and the 15 mg/kg/q8h IV group, significantly higher than the placebo group (*p* < 0.001 and *p* = 0.001, respectively), regardless of the strain’s azole susceptibility. In Negri et al.’s study, the median survival time was 8 days for the 8 mg/kg/q8h IV group and 9 days for the 15 mg/kg/q8h IV group, with total survival rates of 52.5% and 69%, respectively, while the median survival time was 2 days and the total survival rate 0% for the placebo group (*p not mentioned*). The histological results on day 3 for both studies showed a reduction in fungal burden for the 15 mg/kg/q8h groups compared to the placebo group. Lackner et al. compared the efficacy of oral and intravenous olorofim in a neutropenic murine model of systemic *A. terreus* infection. Survival on day 10 post-infection was significantly higher in the treated groups compared to the control group, with a 100% survival rate for the IV group and 90% for the oral group (*p* ≤ 0.0001). The team assessed renal fungal burden on day 10 through culture and found a log_10_ reduction of 1.99 for the IV group and 1.07 for the oral group compared to the placebo group (*p* ≤ 0.0001).

Seyedmousavi et al. established two distinct animal models: a neutropenic murine model of systemic *A. fumigatus*, *A. nidulans*, and *A. tanneri* infection and a murine model of chronic granulomatous disease (CGD) infected through the pulmonary route with the same strains [26]. In both models, olorofim was administered intraperitoneally at a dose of 15 mg/kg/q8h. The efficacy was evaluated against placebo based on survival on day 10, serum GM levels on day 3 and day 10, fungal burden and renal histology on day 3 for the neutropenic model, and fungal burden and pulmonary histology on day 3 for the CGD model. The survival on day 10 appeared to be better in the treated group in both models (*p not mentioned*). Serum GM levels on day 3 seemed lower in the treated groups than in the placebo groups (*p not mentioned*). Only groups infected with *A. tanneri* showed serum GM levels on day 3 similar to those of the controls, with a decrease observed on day 10. Renal and pulmonary fungal burdens were significantly lower in the treated groups, regardless of the strain. Renal and pulmonary histology on day 3 revealed fewer fungal elements and inflammatory lesions in the treated groups compared to the placebo group. This same team conducted studies in neutropenic murine models of systemic scedosporiosis and lomentosporiosis [27]. The efficacy of a 15 mg/kg/q8h dose was evaluated against placebo based on survival on day 10, serum 1,3-β-D-glucan (BD) levels on day 3, and renal fungal burden and histology on day 3. The overall survival seemed better in the olorofim-treated group compared to the control group (*p not mentioned*). Serum BD levels and renal fungal burden were significantly lower in the treated groups compared to the control groups.

Furthermore, olorofim’s efficacy was tested in animal models of dermatophytosis. Mirbzadeh et al. created an immunosuppressed model of guinea pigs through corticosteroid therapy and infected them with a *Microsporum gypseum* strain [28]. Olorofim was topically administered to the created lesions, and the scales were analyzed by optical microscopy on day 7 of treatment. Absence of fungal elements and clinical improvement were observed in the treated group, whereas cutaneous lesions and alopecia were still present in the control group.

Lastly, Wiederhold et al. studied olorofim’s in vivo efficacy against dimorphic fungi in a murine model of central nervous system coccidioidomycosis [23]. In one group, efficacy was evaluated through survival on day 30 after a 14-day treatment with oral doses of 20 or 40 mg/kg/day in two or three administrations, while in another group, it was based on brain fungal burden on day 9 for all, and on death or on day 30 for survivors, after a 7-day treatment with the same doses. In the “survival group”, the median survival time for the treated group was significantly higher than the placebo group (*p* ≤ 0.0001). However, upon treatment discontinuation on day 14, rapid mortality occurred for mice receiving olorofim twice daily, regardless of the dose, with no significant difference in overall survival on day 30 compared to the placebo group. When administered three times daily, the median survival time for the treated group was significantly higher than the placebo group (*p* ≤ 0.0001) and was maintained throughout day 30 for the daily dose of 40 mg/kg (13 mg/kg/q8h). When comparing the fractioning into two or three administrations for a daily dose of 40 mg/kg, a significant difference in median survival time and overall survival was not established despite an apparent trend in favor of the three administrations (*p* = 0.06). In the “fungal burden group”, the quantity of fungi on day 9 was significantly lower in mice treated with fluconazole 25 mg/kg × 2/day compared to the placebo group (*p* ≤ 0.0001), and in those treated with olorofim, regardless of the dose and frequency of administration, with a very low fungal burden in the group receiving 40 mg/kg in three administrations (*p* ≤ 0.01). At the end of treatment, a re-ascension of the fungal burden was observed immediately after discontinuation of fluconazole and olorofim, with no significant difference compared to the placebo, except for mice on the olorofim regimen of 40 mg/kg in three administrations, where a significant decrease in fungal burden was maintained (*p* ≤ 0.0001).

In humans, published data are more limited. Among the 18 phase I clinical trials registered on clinicaltrials.gov, only one study’s results are available. It is a randomized, double-blind, placebo-controlled trial in 40 healthy male volunteers [29]. The primary objective was to determine the tolerability of olorofim at increasing doses, the main outcome measure being the occurrence of adverse events. The all-cause mortality was 0%. Overall, regardless of the dose administered, one out of six men experienced minor adverse events such as headaches, eczema, or epistaxis. The study’s pharmacokinetic and pharmacodynamic data are not published.

**Table 2:** Preclinical and clinical studies of olorofim’s pharmacokinetics and pharmacodynamics.

Reference Study Type	Population	OLR Dose Route	PK/PD	Safety Tolerability
[11] Preclinical	neutropenic CD-1 male mice infected by an *Aspergillus terreus* strain (OLR MIC = 0.008 mg/L)	10 mg/kg/q12h orally or IV	C_min_ orally = 0.8 mg/L C_min_ IV = 0.3 mg/L AUC_0–24h_ ≈ 22.5 mg·h/L	No adverse events
[24] Preclinical	neutropenic CD-1 male mice infected by wild-type and TR_34_/L98H *Aspergillus fumigatus* strains (OLR MIC = 0.03 mg/L)	24 mg/kg/q24h or 8 mg/kg/q8h or 15 mg/kg/q8h IV	Linear PK between 4 and 15 mg/kg/q8h; Protein binding ≈ 99%; Time-dependent antifungal effect: - Dose fractionation studies revealed a reduction in serum GM when administered every 12 h and a total suppression of serum GM when administered every 8 h (*p* = 0.028 and *p* = 0.024 respectively) - PK indexes studies revealed a correlation between C_min_/MIC and the effect of OLR (assessed by serum GM at the end of the experiment and the area under GM-time curve), as well as the fraction of the dosing interval during which the total plasma concentration was above the MIC T > MIC 1 mg/L Target reduction of 27% of the AUC-GM was achieved for C_min_/MIC = 9.1; therefore C_min_ = 0.27 mg/L	No adverse events *
[25] Preclinical	neutropenic CD-1 male mice infected by *Aspergillus flavus* strains (OLR MIC = 0.03 mg/L)	24 mg/kg/q24h or 8 mg/kg/q8h or 15 mg/kg/q8h IV	Serum GM total suppression for C_min_ = 0.3 mg/L and C_min_/MIC ≈ 10 in the invasive sinusitis cellular model and near-total suppression for 15 mg/kg/q8h in the sinopulmonary aspergillosis murine model; 27% reduction in serum GM comparable to that of a posaconazole AUC = 47 mg·h/L achieved for a mean C_min_/MIC = 13.38 [9,10,11,12,13,14,15,16,17,18,19]	NM
[26] Preclinical	neutropenic CD-1 female mice infected by a wild-type *A. fumigatus* strain (OLR MIC = 0.008 mg/L)	2.5–5–10–15–20 mg/kg single dose IP	Linear PK between 2.5 and 20 mg/kg; Area under the concentration–time curve linearly correlated to the dose (*R* = 0.96); T_max_ reached within 0.5 h between 2.5 and 15 mg/kg; C_min_ above the efficacy level required as seen on other murine models for doses 10, 15 and 20 mg/kg	No adverse events
[29] Phase I double-blind, randomized, placebo-controlled clinical trial	40 healthy male volunteers aged between 18 and 45 years divided into 5 cohorts of 8	0.25–0.75–1.5–3–4 mg/kg single dose IV	NM	Serious AE: 0% Minor AE: 16.67% (i.e., epistaxis, paresthesia, headache, eczema)

* Preliminary studies were conducted to determine the maximal tolerated dose. AE: adverse events. AUC: area under the concentration–time curve. AUC-GM: area under the serum galactomannan–time curve. C_max_: maximal plasmatic concentration. C_min_: minimal plasmatic concentration. GM: galactomannan. IP: intraperitoneal. IV: intravenous. NM: not mentioned. OLR: olorofim. PD: pharmacodynamics. PK: pharmacokinetics. PSC: posaconazole. T_max_: time needed to reach the C_max_ after administration.

**Table 3:** Preclinical studies evaluating the efficacy of olorofim in animal models of invasive fungal infection.

Reference	Infection Model Fungus (OLR MIC) Population	Control	OLR Route OLR Dose TT Duration	Measurement *(Method)*	Efficacy
[11]	Invasive systemic infection through IV inoculation in a neutropenic murine model *Aspergillus terreus* (0.008 mg/L) 10 male neutropenic CD-1 mice per group	Placebo IV excipient 1 mg/kg/day IV Amphotericin B	Oral or IV 10 mg/kg/q12h 9 days	Survival on day 10 *(log-rank test)* Renal fungal burden on death or on day 10 *(CFU)*	**Survival on day 10 vs. placebo** Better survival rates in OLR-treated mice than in placebo-treated mice (*p* ≤ 0.0001). No significant difference in survival rates between the IV and oral route.
					**Renal fungal burden on day 10** Reduction in fungal burden in OLR-treated mice as compared to placebo-treated mice (*p* ≤ 0.0001).
[23]	CNS infection in a murine model *Coccidioides immitis* (0.016 mg/L) 10 male ICR mice per group	Placebo oral excipient 25 mg/kg × 2/day oral fluconazole	Oral 20 mg/kg/day in 2 or 3 administrations 40 mg/kg/day in 2 or 3 administrations 14 days	Survival on day 30 *(log-rank test)*	**Survival on day 30 vs. placebo** Significantly higher median survival time in OLR- and FLC-treated mice than in placebo-treated mice (*p* ≤ 0.0001), with no significant difference in the total survival rate compared to placebo for mice treated with OLR 20 mg/kg in 2 or 3 administrations nor mice treated with OLR 40 mg/kg in 2 administrations. Mice treated with OLR 40 mg/kg in 3 administrations presented the highest total survival rate (*p* = 0.0007).
[23]	CNS infection in a murine model *Coccidioides immitis* (0.016 mg/L) 10 male ICR mice per group	Placebo oral excipient 25 mg/kg × 2/day oral fluconazole	Oral 20 mg/kg/day in 2 or 3 administrations 40 mg/kg/day in 2 or 3 administrations 14 days	Brain fungal burden on day 9 *(CFU)* Brain fungal burden at death or on day 30 for survivors *(CFU)*	**Brain fungal burden on day 9 vs. placebo** Significant log_10_ reduction of median fungal burden in mice treated with FLC and OLR 40 mg/kg (*p* ≤ 0.0001) as compared to placebo-treated mice and other OLR regimens.
					**Brain fungal burden on day 30 vs. placebo** Only mice treated with OLR 40 mg/kg maintained a significant log_10_ reduction of the median fungal burden on day 30. Mice treated with FLC or other OLR regimens showed no significant differences with placebo-treated mice.
[25]	Invasive sinopulmonary infection through nasal inoculation in a neutropenic murine model *Aspergillus flavus* 4 strains (0.03 mg/L) 10 male neutropenic CD-1 mice per group, per strain	Placebo IV excipient 20 mg/kg/day oral posaconazole	IV 24 mg/kg/q24h or 8 mg/kg/q8h or 15 mg/kg/q8h 3 days	Survival on day 10 *(log-rank test)* Pulmonary histology on day 3 *(GMS)*	**Survival on day 10 vs. placebo** Better apparent median survival time and total survival rates than placebo for OLR doses of 8 mg/kg/q8h and 15 mg/kg/q8h *(p not mentioned)*.
					**Pulmonary histology on day 3 vs. placebo** Few or no fungal elements in OLR-treated mice as compared to placebo-treated mice, where severe inflammation, necrosis, hemorrhage, edema, necrotizing vasculitis, vascular invasion, and thrombosis were observed.
[26]	Invasive systemic infection through IV inoculation in a neutropenic murine model *Aspergillus fumigatus* (0.008 mg/L) *Aspergillus nidulans* (0.008 mg/L) *Aspergillus tanneri* (0.06 mg/L) 17 female neutropenic CD-1 mice per group: 10 for survival study, 3 for GM measurement and histology, 4 for fungal burden measurement	Placebo IP PBS	IP 15 mg/kg/q8h 9 days	Survival on day 10 *(log-rank test)* Serum GM On day 3 and day 10 *(EIA)* Renal fungal burden on day 3 *(qpCR)* Renal histology on day 3 *(GMS)*	**Survival on day 10 vs. placebo** Better apparent survival rates independently of the strain in OLR-treated mice as compared to placebo-treated mice *(p not mentioned)*.
					**Serum GM on day 3 vs. placebo** Lower serum GM levels in OLR-treated mice than in placebo-treated mice *(p not mentioned)*.
					**Renal fungal burden on day 3 vs. placebo** Three- to six-fold significant reduction of the mean fungal burden in OLR-treated mice as compared to placebo-treated mice *(p* ≤ 0.0001 in the *A. fumigatus* model, *p* ≤ 0.05 in the *A. nidulans* and *A. tanneri* models).
					**Renal histology on day 3 vs. placebo** Few or no fungal elements in OLR-treated mice as compared to placebo-treated mice, where abundant hyphae, severe inflammatory infiltrations, and necrosis were observed.
[26]	Invasive systemic infection through inhalation in a CGD murine model *Aspergillus fumigatus* (0.008 mg/L) *Aspergillus nidulans* (0.008 mg/L) *Aspergillus tanneri* (0.06 mg/L) 17 male *gp91^-/-^ phox* CD-1 mice per group: 10 for survival study, 3 for GM measurement and histology, 4 for fungal burden measurement	Placebo IP PBS	IP 15 mg/kg/q8h 9 days	Survival on day 10 *(log-rank test)* Serum GM On day 3 and day 10 *(EIA)* Pulmonary fungal burden on day 3 *(qpCR)* Pulmonary histology on day 3 *(GMS)*	**Survival on day 10 vs. placebo** Better apparent survival rates independently of the strain in OLR-treated mice as compared to placebo-treated mice *(p not mentioned)*.
					**Serum GM on day 3 vs. placebo** Lower serum GM levels in OLR-treated mice than in placebo-treated mice *(p not mentioned)*.
					**Pulmonary fungal burden on day 3 vs. placebo** Eight- to twenty-two-fold significant reduction in the mean fungal burden in OLR-treated mice as compared to placebo-treated mice *(p* ≤ 0.01 in the *A. fumigatus* model, *p* ≤ 0.001 in the *A. nidulans* model, *p* ≤ 0.0001 in the *A. tanneri* model).
					**Pulmonary histology on day 3 vs. placebo** Few or no fungal elements in OLR-treated mice as compared to placebo-treated mice, where abundant hyphae and extensive necrotic granulomas were observed.
[26]	Invasive systemic infection through inhalation in a CGD murine model *Aspergillus fumigatus* (0.008 mg/L) *Aspergillus nidulans* (0.008 mg/L) *Aspergillus tanneri* (0.06 mg/L) 14 male *gp91^-/-^ phox* CD-1 mice per group	Placebo IP PBS 20 mg/kg/day IP voriconazole	IP 15 mg/kg/q8h 9 days	Survival on day 10 *(log-rank test)* Pulmonary histology on day 3 *(HE)*	**Survival on day 10 vs. placebo** Better apparent survival rates independently of the strain in OLR-treated mice and VOR-treated mice as compared to placebo-treated mice *(p not mentioned)*. VOR-treated mice seemed to have a better survival rate than OLR-treated mice in the *A. fumigatus* model. In the *A. nidulans* and *A. tanneri* models, OLR-treated mice seemed to have a better survival rate than VOR-treated mice.
					**Pulmonary histology on day 3 vs. placebo** Few or no fungal elements in OLR-treated mice as compared to placebo-treated mice, where abundant hyphae and extensive necrotic granulomas were observed. Few or no fungal elements in VOR-treated mice, except in the *A. tanneri* model, where abundant hyphae and extensive necrotic granulomas were observed, as in the placebo-treated mice.
[27]	Invasive systemic infection through IV inoculation in a neutropenic murine model *Scedosporium apiospermum* (0.016 mg/L) *Scedosporium boydii* (0.016 mg/L) *Lomentospora prolificans* (0.03 mg/L) 17 female neutropenic CD-1 mice per group: 10 for survival study, 3 for BD measurement and histology, 4 for fungal burden measurement	Placebo IP PBS	IP 15 mg/kg/q8h 9 days	Survival on day 10 *(log-rank test)* Serum BD on day 3 *(colorimetric assay)* Renal fungal burden on day 3 *(qpCR)* Renal histology on day 3 *(GMS)*	**Survival on day 10 vs. placebo** Better apparent survival rates independently of the strain in OLR-treated mice as compared to placebo-treated mice *(p not mentioned)*.
					**Serum BD on day 3 vs. placebo** Significantly lower serum BD levels in OLR-treated mice than in placebo-treated mice (*p* ≤ 0.001 in the *S. apiospermum* and *S. boydii* models, *p* ≤ 0.0001 in the *L. prolificans* model).
					**Renal fungal burden on day 3 vs. placebo** Four- to six-fold significant reduction in the mean fungal burden in OLR-treated mice as compared to placebo-treated mice *(p* ≤ 0.0001 in the *S. apiospermum* and *S. boydii* models, *p* ≤ 0.01 in the *L. prolificans* model).
					**Renal histology on day 3 vs. placebo** Few or no fungal elements in OLR-treated mice as compared to placebo-treated mice, where abundant hyphae and extensive necrotic granulomas were observed.
[28]	Dermatophytosis in an immunosuppressed guinea pig model *Microsporum gypseum* (0.03 mg/L) 9 albino female guinea pigs immunosuppressed by corticosteroids, divided into 3 groups	Placebo topical PEG300 1% topical clotrimazole	Topical 100 µL of 0.1 mg/mL of olorofim in PEG300 7 days	Surface scrapings from inoculation site *(optical microscopy)*	**Scrapings on day 7 of TT vs. placebo** No fungal elements in OLR-treated guinea pigs as compared to placebo-treated guinea pigs, which presented persistent alopecia and skin lesions. OLR-treated guinea pigs also presented an apparent reduction in skin lesions and faster capillary regrowth in comparison to CTZ-treated guinea pigs.

AMB: amphotericin B. BD: 1,3-β-D-glucan. CFU: colony-forming unit. CGD: chronic granulomatous disease. CNS: central nervous system. CTZ: clotrimazole. EIA: enzyme immunoassay. FLC: fluconazole. GMS: Grocott–Gömöri Methenamine Silver stain. HE: Hematoxylin–Eosin stain. IP: intraperitoneal. IV: intravenous. MIC: minimal inhibitory concentration. NM: not mentioned. OLR: olorofim. PBS: phosphate-buffered saline. qPCR: quantitative real-time polymerase chain reaction. PSC: posaconazole. TT: treatment. VOR: voriconazole. WT: wild-type.

## 4. Discussion

The data on olorofim’s efficacy against various fungal species, especially *A. fumigatus*, revealed consistently low MICs, regardless of azole resistance. Cryptic species within the *Fumigati* complex, like *A. lentulus*, *A. udagawae*, and *A. thermomutatus*, showed similarly low MICs, significantly below the epidemiological cut-off of 0.25 mg/L suggested for wild-type *A. fumigatus* by Jørgensen et al. [7]. Olorofim also displayed notable effectiveness against species beyond the *Fumigati* complex. Even notoriously resistant strains, such as those from the *Usti* complex, showcased relatively low MICs. However, some *Aspergillus* species from the *Chevalieri* clade exhibited high MICs, indicating variability. Furthermore, olorofim was tested against various filamentous fungi encountered in human pathology, demonstrating efficacy against certain strains from resistant genera like *Scedosporium*, *Lomentospora*, *Rasamsonia,* and *Talaromyces* but weak activity against dematiaceous fungi whose melanin constitutes a formidable protection against outside aggressions. While promising against dermatophytes, notably against *T. indotineae*, additional research involving a more extensive strain set is necessary for conclusive insights into olorofim’s efficacy. It displays a unique range of action, effectively combatting numerous filamentous and dimorphic fungi while showing inactivity against yeasts and Mucorales. This distinct spectrum of activity becomes clearer by considering the phylogenetics of its target, the fungal DHODH enzyme. Olorofim selectively inhibits some class 2 DHODH enzymes, notably in *Aspergillus* spp., while other class 2 DHODH enzymes, such as those in *Candida* spp. and *Cryptococcus* spp., remain unaffected due to nucleotide modifications throughout the years affecting the enzyme’s active site [3]. Additionally, the inability to affect class 1A DHODH in some Mucorales may explain olorofim’s natural inefficacy against this fungal group [30].

Interestingly, the simultaneous use of azoles alongside olorofim could lead to inefficacy, as an antagonism was observed in *A. fumigatus* by the growth of colonies within the olorofim inhibition zone in contact with voriconazole [31]. Investigations on the MFIG001 strain revealed that concentrations of itraconazole below the MIC led to the upregulation of genes in the de novo pyrimidine synthesis pathway, whereas downregulation occurred when concentrations exceeded the MIC, suggesting metabolic disruption. The identification of two transcription factors, HapB and AreA, linked to both azole and olorofim resistance, indicates the complexity and interconnection of both pathways, with a possibility of cross-resistance due to mutations in shared transcription factor genes.

The possible emergence of resistant strains always poses a significant concern when dealing with antimicrobial drugs. Under specific circumstances, resistance to olorofim can develop, as observed in a study by Buil et al., where 976 *A. fumigatus* patient-derived strains were inoculated on olorofim-containing agar [32]. A single colony from one strain showed olorofim resistance, indicating potential mutation acquisition. Further investigation of *A. fumigatus* strains supported this phenomenon, displaying an estimated frequency of spontaneous mutations between 10^−7^ and 10^−9^. Sequencing these colonies revealed a predominant substitution mutation at the G119 locus, reducing olorofim’s binding to DHODH’s active site and leading to resistance. These findings stress the importance of avoiding agricultural compounds like ipflufenoquin, an agricultural DHODH inhibitor, that could potentially induce cross-resistance [33]. They also underline the need for a collaborative “One Health” approach between mycology societies and agricultural fungicide developers to maintain a balance between human health and environmental preservation.

The main limit of this review is the lack of published data on humans, particularly in terms of pharmacokinetics and pharmacodynamics. Metabolism, elimination, drug–drug interactions, and individual variability data have not been entirely published to date despite the completion of 18 phase I studies. A phase I clinical trial on 24 healthy male volunteers showed olorofim being rapidly cleared from the plasma compartment with a volume of distribution of 3 L/kg [34]. Steady rate concentrations were reached 24 h after a loading intravenous dose of 4 mg/kg twice daily and a maintenance intravenous dose of 2.5 mg/kg twice daily. Trough concentrations ≥ 0.7 µg/mL were reached for all by day 2. Terminal elimination half-life ranged from 20 to 30 h, with secondary concentration peaks observed during this phase, indicating possible enterohepatic recirculation. Another phase I clinical trial on healthy male and female volunteers showed steady concentrations and trough levels ≥ 0.7 µg/mL within 3 days after a daily oral intake of 360 mg of olorofim [35]. Secondary peaks during the elimination phase were also observed. Moreover, an open-label study showed increased concentrations of midazolam when administered with olorofim, indicating the latter is a weak CYP3A4 inhibitor [36]. Lastly, multiple unpublished case reports have demonstrated the effectiveness of olorofim in treating lomentosporiosis and disseminated coccidioidomycosis [37]. The results of a phase II clinical trial on the efficacy of olorofim in difficult-to-treat filamentous fungal infections (aspergillosis, including azole-resistant; scedosporiosis, lomentosporiosis, coccidioidomycosis, and *Scopulariopsis* IFIs) in patients with limited treatment options were recently presented and are very promising [38]. In a population of 203 patients, complete or partial response was obtained in 36% by day 42 and 34.2% by day 84, excluding patients with coccidioidomycosis due to the inability of proving fungal burden eradication by day 84. Meanwhile, stable disease was achieved in 75.2% by day 42 and in 63.4% by day 84. All-cause mortality was 11.4% and 15.8%, respectively. Safety-wise, olorofim was generally well tolerated, even in patients having received therapy for more than 2 years, in line with what has been observed in several case reports. Only 9.9% of patients presented altered hepatic function possibly due to olorofim, leading to permanent discontinuation of drug administration in only 2.5% of patients. The results of the ongoing phase III clinical trial on the efficacy of olorofim vs. liposomal amphotericin B in the treatment of invasive aspergillosis are eagerly awaited to further evaluate olorofim’s clinical impact.

## 5. Conclusions

This review has compiled MIC results for approximately 2100 strains of *Aspergillus* of all species, 670 strains of other filamentous fungi that have become increasingly prevalent in human pathology, and around 160 strains of dimorphic fungi, all isolated from patients around the globe. The review has also gathered in vivo data on the pharmacokinetics, pharmacodynamics, efficacy, and tolerance of olorofim in the treatment of invasive fungal infections in animal models. Access to the PK-PD data on humans and results from the ongoing phase III clinical trial will provide for a better understanding of the relationship between olorofim, its fungal target, and the human organism.

## Supplementary Materials

The following supporting information can be downloaded at: www.mdpi.com/article/10.3390/jof10050345/s1, Table S1: Preclinical and clinical studies of olorofim’s pharmacokinetics and pharmacodynamics. Table S2: Preclinical studies evaluating the efficacy of olorofim in animal models of invasive fungal infection.
