# Supplementary material for: The Role of Olorofim in the Treatment of Filamentous Fungal Infections: A Review of In Vitro and In Vivo Studies
Source: J Fungi (Basel). 2024 May 10;10(5):345. doi: 10.3390/jof10050345 (PMC11121921; doi:10.3390/jof10050345)
Supplement: Supplementary file 1 [file jof-10-00345-s001.zip › jof-2970961-supplementary.pdf]

Journal of Fungi

MDPI

**Supplementary Table S1.** Preclinical and clinical studies of olorofim's pharmacokinetics and pharmacodynamics

| Reference<br>(Study type) | Population | ORL<br>Measurement time | OLR dose<br>Route | PK/PD | Safety<br>Tolerability |
|---|---|---|---|---|---|
| Hope *et al.*<br>*mBio* 2017<br>(Preclinical) | neutropenic CD-1 male mice infected by:<br><br>*Aspergillus fumigatus*<br>$TR_{34}$/L98H<br>(OLR MIC = 0.03 mg/L)<br><br>*Aspergillus fumigatus*<br>wild-type<br>(OLR MIC = 0.03 mg/L) | Before administration<br><br>After administration:<br>H2<br>H4<br>H8<br><br>3 mice per dose and per measurement time | 24 mg/kg/q24h<br>IV<br><br>8 mg/kg/q8h<br>IV<br><br>15 mg/kg/q8h<br>IV | Linear PK between 4-15 mg/kg/q8h<br>Protein binding ≈ 99%<br><u>Dose-fractionation study *vs.* placebo:</u><br>*PD parameter: serum GM at 78 hours*<br>24 mg/kg/q24h: no significant difference<br>12 mg/kg/q12h: serum GM reduction *(P = 0,028)*<br>8 mg/kg/q8h: serum GM total suppression *(P = 0,024)*<br>independent of azole susceptibility<br>→ time-dependent antifungal effect<br><u>PK indexes study:</u><br>*PD parameters: serum GM at 78 hours, AUC-GM 0-78 hours*<br>$C_{max}$/MIC: no correlation<br>AUC/MIC: no correlation<br>$C_{min}$/MIC: $r^2$ = 0.983 *(serum GM)* and 0.998 *(AUC-GM)*<br>T > MIC 1 mg/L: $r^2$ = 0.996 *(serum GM)* and 1.00 *(AUC-GM)*<br>independent of azole susceptibility<br>→ time-dependent antifungal effect<br><u>$C_{min}$/MIC, serum GM and AUC-GM:</u><br>*Target: 27% reduction of the AUC-GM*<br>$C_{min}$/MIC = 9.1<br>$C_{min}$ = 0.27 mg/L | No adverse events* |
| Negri *et al.*<br>*J Infect Dis* 2018<br>(Preclinical) | neutropenic CD-1 male mice infected by 4 *Aspergillus flavus* strains<br>(OLR MIC = 0.03 mg/L) | Before administration<br><br>After administration:<br>between 6-78 hours<br><br>3 mice per dose and per measurement time | 24 mg/kg/q24h<br>IV<br><br>8 mg/kg/q8h<br>IV<br><br>15 mg/kg/q8h<br>IV | <u>Invasive sinusitis cellular model:</u><br>*PD parameter: GM*<br>GM total suppression:<br>$C_{min}$ = 0.3 mg/L<br>$C_{min}$/MIC ≈ 10<br><u>Sinopulmonary aspergillosis murine model:</u><br>*PD parameters: serum GM at 78 hours, AUC-GM*<br>Near-total suppression of serum GM for 15 mg/kg/q8h<br>Reduction in serum GM comparable to a posaconazole AUC of 47 mg·h/L:<br>$C_{min}$/MIC = [9-19], mean = 13.38 | NM |
| Lackner *et al.*<br>*J Antimicrob Chemother* 2018<br>(Preclinical) | neutropenic CD-1 male mice infected by an *A. terreus* strain<br>(OLR MIC = 0.008 mg/L) | NM | 10 mg/kg/q12h<br>orally or IV | $C_{min}$ VO = 0.8 mg/L<br>$C_{min}$ IV = 0.3 mg/L<br>$AUC_{0-24h}$ ≈ 22.5 mg·h/L | No adverse events |
| Seyedmousavi *et al.*<br>*Antimicrob Agents Chemother* 2019 | neutropenic CD-1 female mice infected by a wild-type *A. fumigatus* strain<br>(OLR MIC = 0.008 mg/L) | Before administration<br><br>After administration:<br>H0.5<br>H1<br>H2<br>H4<br>H8<br><br>2 mice per dose and per measurement time | 2.5 mg/kg<br>single dose<br>IP<br>5 mg/kg<br>single dose<br>IP<br>10 mg/kg<br>single dose<br>IP<br>15 mg/kg<br>single dose<br>IP<br>20 mg/kg<br>single dose<br>IP | Linear PK between 2.5-20 mg/kg<br>R = 0.96 *(AUC)*<br><br>$C_{max}$ = 1.345 mg/L<br>$C_{min}$ = 0.185 mg/L<br>$T_{max}$ = 0.5 h<br>$AUC_{0-8h}$ = 4.399 mg·h/L<br><br>$C_{max}$ = 2.860 mg/L<br>$C_{min}$ = 0.122 mg/L<br>$T_{max}$ = 0.5 h<br>$AUC_{0-8h}$ = 8.478 mg·h/L | No adverse events |

| | | | | | |
|---|---|---|---|---|---|
| | | | | $C_{max}$ = 5.220 mg/L<br>$C_{min}$ = 0.986 mg/L<br>$T_{max}$ = 0.5 h<br>$AUC_{0-8h}$ = 16.840 mg·h/L | |
| | | | | $C_{max}$ = 5.280 mg/L<br>$C_{min}$ = 1.560 mg/L<br>$T_{max}$ = 0.5 h<br>$AUC_{0-8h}$ = 27.244 mg·h/L | |
| | | | | $C_{max}$ = 4.000 mg/L<br>$C_{min}$ = 2.355 mg/L<br>$T_{max}$ = 2 h<br>$AUC_{0-8h}$ = 28.475 mg·h/L | |
| Dr. Salvatore Febbraro 2015 (Phase I double-blind, randomized, placebo-controlled clinical trial) | 40 healthy male volunteers aged between 18 and 45 years divided into 5 cohorts of 8 | Before administration<br>During infusion: H1 - H2 - H3 - H4<br>After infusion: H4.5 - H5 – H5.5 - H6 - H7 - H8 - H10 - H12 - H24 - H36 - H48 - H72 - H96 - H120 | 0.25 mg/kg single dose IV | NM | Serious AE: 0%<br>Other AE: epistaxis 16.67% *vs.* 50% placebo |
| | | | 0.75 mg/kg single dose IV | | Serious AE: 0%<br>Other AE: paresthesia 16.67% *vs.* 0% placebo |
| | | | 1.5 mg/kg single dose IV | | Serious AE: 0%<br>Other AE: headaches 16.67% *vs.* 0% placebo |
| | | | 3 mg/kg single dose IV | | Serious AE: 0%<br>Other AE: eczema 16.67% *vs.* 0% placebo |
| | | | 4 mg/kg single dose IV | | Serious AE: 0%<br>Other AE: epistaxis 16.67% *vs.* 0% placebo |

*Preliminary studies were conducted to determine the maximal tolerated dose.
AE: adverse events. AUC: area under the concentration-time curve. AUC-GM: area under the serum galactomannan-time curve. $C_{max}$: maximal plasmatic concentration. $C_{min}$: minimal plasmatic concentration. GM: galactomannan. IP: intraperitoneal. IV: intravenous. NM: not mentioned. OLR: olorofim. PD: pharmacodynamics. PK: pharmacokinetics. PSC: posaconazole. $T_{max}$: time needed to reach the $C_{max}$ after administration.

**Supplementary Table S2.** Preclinical studies evaluating the efficacy of olorofim in animal models of invasive fungal infection.

| Reference | Infection model<br>Fungus<br>(OLR MIC)<br>Population | Control | OLR route<br>OLR dose<br>TT duration | Measurement<br>*(method)* | Efficacy *(P)* |
|---|---|---|---|---|---|
| Hope *et al.*<br>*mBio* 2017 | Invasive pulmonary infection through nasal inoculation in a neutropenic murine model<br><br>*Aspergillus fumigatus*<br>2 WT strains<br>(0.03 mg/L)<br>2 azole-resistant strains<br>(0.03 mg/L)<br><br>10 male neutropenic CD-1 mice per group, per strain | Placebo<br>IV excipient<br><br>10 mg/kg/day oral posaconazole | IV<br><br>24 mg/kg/q24h<br>8 mg/kg/q8h<br>15 mg/kg/q8h<br><br>3 days | Survival on day 10<br>*(log-rank test)*<br><br>Pulmonary histology on day 3<br>*(GMS)* | **Survival on day 10 *vs.* placebo**<br>OLR 24 mg/kg/q24h, all strains *(>0.05)*<br>Median survival time = 3.5 days<br>Total survival rate = 17.5%<br>OLR 8 mg/kg/q8h, all strains *(<0.001)*<br>Median survival time ≈ 8 days<br>Total survival rate = 67.5%<br>OLR 15 mg/kg/q8h, all strains *(0.001)*<br>Median survival time ≈ 8 days<br>Total survival rate = 60%<br>PSC, azole-susceptible strains *(0.001)*<br>Median survival time > 10 days<br>Total survival rate = 85%<br>PSC, azole-resistant strains *(0.001)*<br>Median survival time = 3 days<br>Total survival rate = 20%<br>Placebo, all strains<br>Median survival time ≈ 3 days<br>Total survival rate = 10%<br>**Pulmonary histology on day 3 *vs.* placebo**<br>OLR 15 mg/kg/q8h<br>Reduction in fungal burden<br>Contained hyphae<br>Minimal vascular lesions<br>PSC<br>Reduction in fungal burden<br>Contained hyphae<br>Placebo<br>Severe inflammation, necrosis<br>Hemorrhage, edema<br>Necrotizing vasculitis, vascular invasion |
| Negri *et al.*<br>*J Infect Dis* 2018 | Invasive sinopulmonary infection through nasal inoculation in a neutropenic murine model<br><br>*Aspergillus flavus*<br>4 strains<br>(0.03 mg/L)<br><br>10 male neutropenic CD-1 mice per group, per strain | Placebo<br>IV excipient<br><br>20 mg/kg/day oral posaconazole | IV<br><br>24 mg/kg/q24h<br>8 mg/kg/q8h<br>15 mg/kg/q8h<br><br>3 days | Survival on day 10<br>*(log-rank test)*<br><br>Pulmonary histology on day 3<br>*(GMS)* | **Survival on day 10 *vs.* placebo**<br>OLR 24 mg/kg/q24h, all strains *(NM)*<br>Median survival time = 4.5 days<br>Total survival rate ≈ 21%<br>OLR 8 mg/kg/q8h, all strains *(NM)*<br>Median survival time ≈ 8 days<br>Total survival rate = 52.5%<br>OLR 15 mg/kg/q8h, all strains *(NM)*<br>Median survival time ≈ 9 days<br>Total survival rate ≈ 69%<br>PSC, all strains *(NM)*<br>Median survival time: NM<br>Total survival rate: NM<br>Placebo, all strains<br>Median survival time = 2 days<br>Total survival rate = 0%<br><br>**Pulmonary histology on day 3 *vs.* placebo**<br>OLR 15 mg/kg/q8h<br>Few or no fungal elements<br>Placebo<br>Severe inflammation, necrosis<br>Hemorrhage, edema |

| | | | | | |
|---|---|---|---|---|---|
| | | | | | Necrotizing vasculitis, vascular invasion, thrombosis |
| Lackner *et al.* *J Antimicrob Chemother* 2018 | Invasive systemic infection through IV inoculation in a neutropenic murine model<br><br>*Aspergillus terreus* (0.008 mg/L)<br><br>10 male neutropenic CD-1 mice per group | Placebo IV excipient<br><br>1 mg/kg/day IV Amphotericin B | Oral or IV<br><br>10 mg/kg/q12h<br><br>9 days | Survival on day 10 *(log-rank test)*<br><br>Renal fungal burden on death or on day 10 *(CFU)* | **Survival on day 10 *vs.* placebo**<br>OLR IV = 100% *(≤0.0001)*<br>OLR oral = 90% *(≤0.0001)*<br>AMB = 0% *(≤0.0001)*<br>Placebo = 10%<br><br>**Renal fungal burden on day 10**<br>OLR IV<br>1.99 $log_{10}$ reduction *vs.* placebo *(≤0.0001)*<br>OLR oral<br>1.07 $log_{10}$ reduction *vs.* placebo *(≤0.0001)* |
| Seyedmousavi *et al.* *Antimicrob Agents Chemother* 2019 | Invasive systemic infection through IV inoculation in a neutropenic murine model<br><br>*Aspergillus fumigatus* (0.008 mg/L)<br>*Aspergillus nidulans* (0.008 mg/L)<br>*Aspergillus tanneri* (0.06 mg/L)<br><br>17 female neutropenic CD-1 mice per group:<br>10 for survival study<br>3 for GM measurement and histology<br>4 for fungal burden measurement | Placebo IP PBS | IP<br><br>15 mg/kg/q8h<br><br>9 days | Survival on day 10 *(log-rank test)*<br><br>Serum GM On day 3 and day 10 *(EIA)*<br><br>Renal fungal burden on day 3 *(qPCR)*<br><br>Renal histology on day 3 *(GMS)* | **Survival on day 10 *vs.* placebo** *(NM)*<br>OLR<br>*A. fumigatus* = 81%<br>*A. nidulans* = 88%<br>*A. tanneri* = 80%<br>Placebo = 10%<br><br>**Serum GM on day 3 *vs.* placebo** *(NM)*<br>OLR<br>*A. fumigatus*:<br>Index = 1 on day 3, index ≈ 0.9 on day 10<br>*A. nidulans*:<br>Index = 1.5 on day 3, index = 0.5 on day 10<br>*A. tanneri*:<br>Index = 3.5 on day 3, index = 1 on day 10<br>Placebo<br>*A. fumigatus* control: index = 3.5 on day 3<br>*A. nidulans* control: index = 3.5 on day 3<br>*A. tanneri* control: index = 3.5 on day 3<br><br>**Renal fungal burden on day 3 *vs.* placebo**<br>OLR<br>*A. fumigatus*:<br>Mean = 5 pg/µg total DNA *(≤0.0001)*<br>*A. nidulans*:<br>Mean = 20 pg/µg total DNA *(≤0.05)*<br>*A. tanneri*:<br>Mean = 50 pg/µg total DNA *(≤0.05)*<br>Placebo<br>*A. fumigatus* control:<br>Mean = 32 pg/µg total DNA<br>*A. nidulans* control:<br>Mean = 60 pg/µg total DNA<br>*A. tanneri* control:<br>Mean = 300 pg/µg total DNA |

| | | | | | |
|---|---|---|---|---|---|
| | | | | | **Renal histology on day 3 *vs.* placebo**<br><u>OLR</u><br>Few or no fungal elements<br><u>Placebo</u><br>Abundant hyphae<br>Severe inflammatory infiltrations<br>Necrosis |
| Seyedmousavi *et al.* *Antimicrob Agents Chemother* 2019 | Invasive systemic infection through inhalation in a CGD murine model<br><br>*Aspergillus fumigatus* (0.008 mg/L)<br>*Aspergillus nidulans* (0.008 mg/L)<br>*Aspergillus tanneri* (0.06 mg/L)<br><br>17 male *gp91*$^{-/-}$ *phox* CD-1 mice per group:<br>10 for survival study<br>3 for GM measurement and histology<br>4 for fungal burden measurement | Placebo<br>IP PBS | IP<br><br>15 mg/kg/q8h<br><br>9 days | Survival on day 10 *(log-rank test)*<br><br>Serum GM On day 3 and day 10 *(EIA)*<br><br>Pulmonary fungal burden on day 3 *(qPCR)*<br><br>Pulmonary histology on day 3 *(GMS)* | **Survival on day 10 *vs.* placebo** *(NM)*<br><u>OLR</u><br>*A. fumigatus* = 88%<br>*A. nidulans* = 75%<br>*A. tanneri* = 63%<br><u>Placebo</u><br>*A. fumigatus* <40%<br>*A. nidulans* = 0%<br>*A. tanneri* = 0%<br><br>**Serum GM on day 3 *vs.* placebo** *(NM)*<br><u>OLR</u><br>*A. fumigatus*:<br>Index = 1.1 on day 3, index ≈ 1 on day 10<br>*A. nidulans*:<br>Index = 2 on day 3, index = 1.7 on day 10<br>*A. tanneri*:<br>Index = 3.2 on day 3, index = 2.8 on day 10<br><u>Placebo</u><br>*A. fumigatus* control: index = 3.5 on day 3<br>*A. nidulans* control: index = 3.5 on day 3<br>*A. tanneri* control: index = 4 on day 3<br><br>**Pulmonary fungal burden on day 3 *vs.* placebo**<br><u>OLR</u><br>*A. fumigatus*:<br>Mean = 0 pg/µg total DNA *(≤0.01)*<br>*A. nidulans*:<br>Mean = 50 pg/µg total DNA *(≤0.001)*<br>*A. tanneri*:<br>Mean = 500 pg/µg total DNA *(≤0.001)*<br><u>Placebo</u><br>*A. fumigatus* control:<br>Mean = 150 pg/µg total DNA<br>*A. nidulans* control:<br>Mean = 400 pg/µg total DNA<br>*A. tanneri* control:<br>Mean = 11,000 pg/µg total DNA<br><br>**Pulmonary histology on day 3 *vs.* placebo**<br><u>OLR</u><br>Few or no fungal elements<br><u>Placebo</u><br>Abundant hyphae<br>Extensive granulomas with necrosis |

| | | | | | |
|---|---|---|---|---|---|
| Seyedmousavi *et al.* *Antimicrob Agents Chemother* 2021 | Invasive systemic infection through IV inoculation in a neutropenic murine model<br><br>*Scedosporium apiospermum* (0.016 mg/L)<br>*Scedosporium boydii* (0.016 mg/L)<br>*Lomentospora prolificans* (0.03 mg/L)<br><br>17 female neutropenic CD-1 mice per group:<br>10 for survival study<br>3 for BD measurement and histology<br>4 for fungal burden measurement | Placebo<br>IP PBS | IP<br><br>15 mg/kg/q8h<br><br>9 days | Survival on day 10 *(log-rank test)*<br><br>Serum BD on day 3 *(colorimetric assay)*<br><br>Renal fungal burden on day 3 *(qPCR)*<br><br>Renal histology on day 3 *(GMS)* | **Survival on day 10 *vs.* placebo** *(NM)*<br>OLR<br>*S. apiospermum* = 80%<br>*S. boydii* = 100%<br>*L. prolificans* = 100%<br>Placebo<br>*S. apiospermum* = 0%<br>*S. boydii* = 20%<br>*L. prolificans* = 20%<br><br>**Serum BD on day 3 *vs.* placebo**<br>OLR<br>*S. apiospermum*: mean = 300 pg/mL *(≤0.001)*<br>*S. boydii*: mean = 100 pg/mL *(≤0.001)*<br>*L. prolificans*: mean ≈ 200 pg/mL *(≤0.0001)*<br>Placebo<br>*S. apiospermum* control: mean = 1,200 pg/mL<br>*S. boydii* control: mean = 1,000 pg/mL<br>*L. prolificans* control: mean = 700 pg/mL<br><br>**Renal fungal burden on day 3 *vs.* placebo**<br>OLR<br>*S. apiospermum*:<br>Mean = 100 pg/µg total DNA *(≤0.0001)*<br>*S. boydii*:<br>Mean = 100 pg/µg total DNA *(≤0.0001)*<br>*L. prolificans*:<br>Mean = 100 pg/µg total DNA *(≤0.01)*<br>Placebo<br>*S. apiospermum* control:<br>Mean = 600 pg/µg total DNA<br>*S. boydii* control:<br>Mean ≈ 475 pg/µg total DNA<br>*L. prolificans* control:<br>Mean ≈ 425pg/µg total DNA<br><br>**Renal histology on day 3 *vs.* placebo**<br>OLR<br>Few or no fungal elements<br>Placebo<br>Abundant hyphae<br>Extensive granulomas with necrosis |
| Mirbzadeh *et al.* *Antimicrob Agents Chemother* 2021 | Dermatophytosis in an immunosuppressed guinea pig model<br><br>*Microsporum gypseum* (0.03 mg/L)<br><br>9 albino female guinea pigs immunosuppressed by corticosteroids, divided into 3 groups | Placebo topical PEG300<br><br>1% topical clotrimazole | Topical<br><br>100 µL of 0.1 mg/mL of olorofim in PEG300<br><br>7 days | Surface scrapings from inoculation site *(optical microscopy)* | **Scrapings on day 7 of TT *vs.* placebo**<br>OLR<br>No fungal elements<br>Reduction in skin lesions<br>Capillary regrowth<br>CTZ<br>No fungal elements<br>Placebo<br>Alopecia<br>Persistent skin lesions |

| | | | | | |
|---|---|---|---|---|---|
| Wiederhold<br>*et al.*<br>*Antimicrob Agents Chemother* 2018 | CNS infection in a murine model<br><br>*Coccidioides immitis*<br>(0.016 mg/L)<br><br>10 male ICR mice per group | Placebo<br>oral excipient<br><br>25 mg/kg x 2/day oral fluconazole | Oral<br><br>20 mg/kg/day in 2 or 3 administrations<br><br>40 mg/kg/day in 2 or 3 administrations<br><br>14 days | Survival on day 30<br>*(log-rank test)* | **Survival on day 30 *vs.* placebo**<br><br><u>OLR 20 mg/kg in 2 administrations</u><br>Median survival time = 22 days *(≤0.0001)*<br>Total survival rate = 10% *(>0.05)*<br><u>OLR 40 mg/kg in 2 administrations</u><br>Median survival time = 26 days *(≤0.0001)*<br>Total survival rate = 30% *(>0.05)*<br><u>FLC</u><br>Median survival time = 30 days *(≤0.0001)*<br>Total survival rate = 50% *(≤0.0001)*<br><u>Placebo</u><br>Median survival time = 9 days<br>Total survival rate = 0%<br><br><u>OLR 20 mg/kg in 3 administrations</u><br>Median survival time = 23.5 days *(≤0.0001)*<br>Total survival rate = 30% *(>0.05)*<br><u>OLR 40 mg/kg in 3 administrations</u><br>Median survival time > 30 days *(≤0.0001)*<br>Total survival rate = 80% *(0.0007)*<br><u>FLC</u><br>Median survival time = 28 days *(≤0.0001)*<br>Total survival rate ≈ 25% *(≤0.0001)*<br><u>Placebo</u><br>Median survival time = 9 days<br>Total survival rate = 0% |
| Wiederhold<br>*et al.*<br>*Antimicrob Agents Chemother* 2018<br>*(cont.)* | CNS infection in a murine model<br><br>*Coccidioides immitis*<br>(0.016 mg/L)<br><br>10 male ICR mice per group | Placebo<br>oral excipient<br><br>25 mg/kg x 2/day oral fluconazole | Oral<br><br>20 mg/kg/day in 2 or 3 administrations<br><br>40 mg/kg/day in 2 or 3 administrations<br><br>14 | Brain fungal burden on day 9<br>*(CFU)*<br><br>Brain fungal burden at death or on day 30 for survivors<br>*(CFU)* | **Brain fungal burden on day 9 *vs.* placebo**<br><br><u>OLR 20 mg/kg in 2 administrations</u><br>Median = 4.36 $\log_{10}$ CFU/g *(≤0.01)*<br><u>OLR 40 mg/kg in 2 administrations</u><br>Median = 3.41 $\log_{10}$ CFU/g *(≤0.01)*<br><u>FLC</u><br>Median = 1.33 $\log_{10}$ CFU/g *(≤0.0001)*<br><u>Placebo</u><br>Median = 5.53 $\log_{10}$ CFU/g<br><br><u>OLR 20 mg/kg in 3 administrations</u><br>Median = 3.28 $\log_{10}$ CFU/g *(≤0.0001)*<br><u>OLR 40 mg/kg in 3 administrations</u><br>Median = 1.95 $\log_{10}$ CFU/g *(≤0.0001)*<br><u>FLC</u><br>Median = 1.33 $\log_{10}$ CFU/g *(≤0.0001)*<br><u>Placebo</u><br>Median = 5.97 $\log_{10}$ CFU/g<br><br>**Brain fungal burden on day 30 *vs.* placebo**<br><br><u>OLR 20 mg/kg in 2 administrations</u><br>Median ≈ 6 $\log_{10}$ CFU/g *(>0.05)*<br><u>OLR 40 mg/kg in 2 administrations</u><br>Median ≈ 5.5 $\log_{10}$ CFU/g *(>0.05)*<br><u>FLC</u><br>Median ≈ 6 $\log_{10}$ CFU/g *(>0.05)*<br><u>Placebo</u><br>Median = 5.67 $\log_{10}$ CFU/g<br><br><u>OLR 20 mg/kg in 3 administrations</u><br>Median ≈ 5.5 $\log_{10}$ CFU/g *(>0.05)*<br><u>OLR 40 mg/kg in 3 administrations</u><br>Median = 1.13 $\log_{10}$ CFU/g *(≤0.0001)*<br><u>FLC</u> |

Median ≈ 6 $log_{10}$ CFU/g *(>0.05)*
Placebo
Median = 5.67 $log_{10}$ CFU/g

AMB: amphotericin B. BD: 1,3-ß-D-glucan. CFU: Colony-Forming Unit. CGD: chronic granulomatous disease. CNS: central nervous system. CTZ: clotrimazole. EIA: enzyme immunoassay. FLC: fluconazole. GMS: Grocott-Gömöri Methenamine Silver stain. HE: Hematoxyline-Eosine stain. IP: intraperitoneal. IV: intravenous. MIC: Minimal Inhibitory Concentration. NM: not mentioned. OLR: olorofim. PBS: Phosphate-Buffered Saline. qPCR: quantitative real-time polymerase chain reaction. PSC: posaconazole. TT: treatment. VOR: voriconazole. WT: wild-type.
